# Risk Stratification in Pulmonary Arterial Hypertension, Update and Perspectives

**DOI:** 10.3390/jcm12134349

**Published:** 2023-06-28

**Authors:** Argyro Vraka, Eleni Diamanti, Mithum Kularatne, Patrick Yerly, Frédéric Lador, John-David Aubert, Benoit Lechartier

**Affiliations:** 1 Pulmonary Division, Lausanne University Hospital, University of Lausanne, 1011 Lausanne, Switzerland; 2 Division of Respiratory Medicine, Department of Medicine, University of Calgary, Calgary, AB T2N 1N4, Canada; 3 Division of Cardiology, Cardiovascular Department, Lausanne University Hospital, University of Lausanne, 1011 Lausanne, Switzerland; 4 Pulmonary Division, Geneva University Hospital, 1211 Geneva, Switzerland

**Keywords:** pulmonary arterial hypertension, risk stratification, PAH prognosis, lung transplantation

## Abstract

Risk stratification in pulmonary arterial hypertension (PAH) is crucial in assessing patient prognosis. It serves a prominent role in everyday patient care and can be determined using several validated risk assessment scores worldwide. The recently published 2022 European Society of Cardiology (ESC)/European Respiratory Society (ERS) guidelines underline the importance of risk stratification not only at baseline but also during follow-up. Achieving a low-risk status has now become the therapeutic goal, emphasising the importance of personalised therapy. The application of these guidelines is also important in determining the timing for lung transplantation referral. In this review, we summarise the most relevant prognostic factors of PAH as well as the parameters used in PAH risk scores and their evolution in the guidelines over the last decade. Finally, we describe the central role that risk stratification plays in the current guidelines not only in European countries but also in Asian countries.

## 1. Introduction 

Pulmonary arterial hypertension (PAH) is a rare disease of high complexity, characterised by pulmonary arterial obstruction leading to progressive right heart failure. PAH encompasses a heterogeneous group of incurable pulmonary vascular disorders that share similar clinical presentations, haemodynamic parameters and therapeutic management strategies [1]. Current PAH therapies target one of the three well-characterised pathways, the nitric oxide (NO), endothelin (ET)-1 and prostacyclin (PGI_2_) signalling pathways [2].

As recommended in the updated 2022 European Society of Cardiology (ESC)/European Respiratory Society (ERS) guidelines for the diagnosis and treatment of pulmonary hypertension (PH) [3], risk stratification is essential for PAH diagnosis in order to evaluate the severity of the disease, determine an appropriate initial treatment strategy and adapt it during routine follow-up assessments [4]. Several multivariable risk assessment scores are described in the literature, notably the score developed by the Swedish Comparative Prospective Registry of Newly Initiated Therapies for PH (COMPERA) [5], the ESC/ERS risk assessment tool [6], the French PH Network Registry score (FPHR) [7], the Registry to Evaluate Early and Long-Term PAH Disease Management (REVEAL)2.0 risk score [8] and the one by the Swedish Pulmonary Arterial Hypertension Register (SPAHR) [3].

The aim of this manuscript is to review the clinical and paraclinical biomarkers that influence PAH prognosis. We also focus on the utility of the main risk stratification tools used in everyday clinical assessment and how they affect PAH patient management, including therapy modification and lung transplantation (LT) referral or listing. We also provide a review of the risk stratification strategies for PAH patients upon diagnosis and during treatment according to the latest guidelines not only in Europe but also worldwide [9].

## 2. PAH Prognostic Factors 

### 2.1. Population Characteristics and Clinical Parameters

PAH prognosis assessment is crucial for defining patient management. Several variables are known to play an important role in the prediction of patient survival. Evaluation of the World Health Organisation (WHO) functional class (FC) upon diagnosis is one of the parameters that may predict a patient’s survival [10]. However, this remains a relatively subjective variable influenced by both patient and physician estimation [11]. Benza et al. [10] and Hoeper et al. [12] found worse survival outcomes in an elderly population compared to a younger one with a similar functional class. Mehari et al. used hospital discharge data from the National Hospital Discharge Survey (NHDS) of the United States of America (USA) to demonstrate a higher rate of hospitalisation in female patients [13].

Signs of right heart failure, rapidity of symptoms’ progression and frequency of syncope are also pertinent clinical parameters with prognostic value. Syncope is usually secondary to cerebral hypoperfusion, caused by autonomic dysfunction and lower adrenergic baroreflex sensitivity in PH patients, leading to a greater susceptibility to systemic hypotension [14]. Right ventricular (RV) dysfunction and an inability to increase cardiac output (CO) represent another pathophysiologic mechanism that explains why syncope is an indicator of disease severity. Occasional episodes of syncope may have no prognostic value but repeated episodes are a putative sign of an increased risk of right heart failure [15]. 

The 6-min walk distance (6MWD) represents a variable of great prognostic value. Achievement of a 6MWD greater than 380–440 m appears to predict better outcomes [5,10]. Improvements by ≥30 m are associated with improved quality of life, justifying the inclusion of 6MWD in stratification and multicomponent endpoints in clinical trials [5,16]. A careful interpretation of the 6MWD values is necessary, since its sensitivity for prognosis prediction may be altered depending on a patient’s characteristics (age, gender) and comorbidities. The usefulness of the 6MWD has been challenged as a primary endpoint in clinical trials due to the lack of correlation between 6MWD changes and survival [17,18]. However, when relying on absolute distance values and not on relative improvement from baseline, this remains a very useful parameter in everyday clinical practice.

Cardiopulmonary exercise testing (CPET) has been widely studied for its role in PH diagnosis and prognosis. Based on the 2022 guidelines, CPET should be considered in symptomatic patients with an intermediate echocardiographic probability of PH in order to further determine PH likelihood [9]. CPET and maximum oxygen uptake (VO2_peak_) measurements may better predict prognosis in PAH than the 6MWD [19]. In PAH and chronic thromboembolic pulmonary hypertension (CTEPH) patients, the ratio of minute ventilation/minute CO_2_ production (V_E_/V_CO2_) ≥ 55 at the anaerobic threshold could predict a poor prognosis at 2 years [20]. 

### 2.2. Biologic and Functional Parameters: Present and Future

N-terminal pro-B-type natriuretic peptide (NT-proBNP) is demonstrated to be an independent predictor of survival [21]. In PAH, as in other forms of PH, increased myocardial stress and right ventricular hypertrophy due to an increase in pulmonary vascular resistance (PVR) may cause a rise in NT-proBNP [22]. A recent systematic review and meta-analysis on PAH patients demonstrated that high levels of NT-proBNP are correlated with a significantly increased risk of mortality or LT in PAH (adjusted pooled HR of 1.19 (1.08–1.32)) [23]. Variation in NT-proBNP levels during idiopathic PAH (iPAH) patient follow-up under treatment is a stronger indicator of transplant-free survival (TFS) than its value at diagnosis [23]. Nickel et al. found that PAH patients with an NT-proBNP < 1800 ng/L during follow-up had the best outcome after 5 years of follow-up [24]. Haddad et al. demonstrated that the NT-proBNP level emerged as a central hub in the many parameters linked to PAH prognosis. When added to a baseline risk model, a serial change in NT-proBNP significantly improved outcome prediction at 5 years [25]. However, when interpreting NT-proBNP values, we have to take into consideration that they can be influenced by several parameters such as age, obesity, left heart disease, renal function or anaemia [26].

Yogaswaran et al. described that g-glutamyl transferase (G-GT), the aspartate aminotransferase/alanine transferase (AST/ALT) ratio and the neutrophil-to-lymphocyte ratio (NLR) can reliably predict survival upon diagnosis and during follow-up. Renal function and its changes upon 6 months of follow-up were also studied and were correlated with survival [27]. Incorporating renal function in the COMPERA risk model may modify the risk evaluation in some patients, particularly those of intermediate risk [27]. Interestingly, gas exchange parameters are not used for the estimation of a patient’s prognosis by any risk assessment tools, whereas their evaluation is relevant in chronic pulmonary vascular diseases. A low partial pressure of arterial blood carbon dioxide (PaCO2) may be a useful noninvasive prognostic factor since a reduced PaCO2 reflects hyperventilation to maintain the partial pressure of arterial blood oxygen (PaO2). Similar results were shown for hypoxemia upon diagnosis as well as during follow-up under PAH medication [28,29,30]. In a recent study, pro-atrial natriuretic peptide (pro-ANP) and high-sensitivity troponin T (hsTnT) were found to have a significant prognostic value in patients with PAH [31]. Under specific PH treatment, a ≥ 3% decrease in arterial oxyhaemoglobin saturation (Sa_O2_) is correlated with poorer survival, independent of ESC/ERS risk stratification [32]. Additionally, diffusing the capacity of the lungs for carbon monoxide (DLCO) has also demonstrated significant transplant-free prognostic information upon diagnosis of PAH [33]. In patients with PAH associated with systemic sclerosis (SSc-PAH), low oxygenated haemoglobin (OxyHem) ≤ 12.5 g/dL upon diagnosis could significantly predict survival (*p* = 0.046), as demonstrated recently by Xanthouli et al. Additionally, a DLCO < 65% correlated with a low OxyHem ≤ 12.5 g/dL could predict PAH prognosis at baseline with 76% sensitivity [34].

There is increasing evidence that inflammation and oxidative stress play a critical role in the pathophysiology of PAH. Although the precise pathophysiological pathways remain obscure in PAH development, the elevation of some plasma cytokines and chemokines such as interleukin (IL)-1α and β, IL-6, IL-8, IL-10, IL-12, chemokine ligand 2 (CCL2), CCL5 and tumour necrosis factor (TNF)-α were correlated with a worse clinical outcome [35]. Boucly et al. reported that three serum cytokines (ß-NGF, CXCL9 and TRAIL) may be potential new prognostic factors of PAH both upon diagnosis and during follow-up [36]. β-NGF and CXCL9 may be predictors of death or LT, whereas high TRAIL levels may be associated with a better prognosis. However, more studies are needed to confirm this statement. Damico et al. demonstrated that high levels of serum endostatin, an angiostatic factor, are associated with disease severity and worse PAH prognosis [37]. Its potential utility as a prognostic biomarker in large prospective cohorts remains to be demonstrated.

Research is underway to provide stronger evidence for the use of proteomics as prognostic factors of PAH [38,39,40]. With proteomic techniques, the profiling of human plasma proteome becomes more feasible in searching for disease-related markers. For example, the expression of chemerin, a protein that induces natural killer cell recruitment into inflamed peripheral tissues, was detected in fibroblasts and pulmonary artery smooth muscle cells in SSc-PAH patients. Chemerin levels were significantly elevated and correlated with PVR in SSc-PAH patients and may be an interesting future biomarker [41]. However, proteomic techniques are not ready to be implemented soon in everyday clinical practice. Furthermore, the assessment of transcriptome patterns in blood has been conducted using unsupervised machine learning in iPAH. Three distinct subgroups were identified with unique blood transcriptomic and clinical features and with different prognoses, supporting the existence of three endo-phenotypes within the iPAH classification. Characteristically, the dysregulation of immunoglobulin genes, *NOG* and *ALAS2* (erythroid ALA-synthase), were the most predictive of the subgroups with the best and worst prognosis, respectively [42].

Finally, Arvidsson et al. investigated the potential relationship between extracellular matrix (ECM)-related proteins and survival in patients with PAH [43]. They demonstrated that high plasma levels of metalloproteinase (MMP)-2, extracted from mixed venous blood samples during right heart catheterisation (RHC), are associated with poor survival. As for other putative biomarkers, prospective studies with validation cohorts are needed to establish the potential clinical applicability.

To summarise, many innovative approaches integrating network medicine and machine learning could potentially be used to identify phenotypes that will better guide risk stratification and target-specific therapeutic approaches in PAH [44].

### 2.3. Haemodynamic and New PH Definition

RHC is the cornerstone procedure for PH diagnosis and classification and is also extremely valuable for follow-up. Several direct and calculated hemodynamic parameters are described to have a relevant prognostic value [9]. In particular, mean pulmonary arterial pressure (mPAP) associated via CO measurement with PVR is undeniably a survival indicator [45].

The definition of precapillary PH was updated in the 6th World Symposium on Pulmonary Hypertension (WSPH) as an mPAP > 20 mmHg at rest and a PVR ≥ 3 WU measured by RHC [46]. The reduction of mPAP from 25 mmHg—compared to the 2015 guidelines—to 20 mmHg was the result of studies reassessing normal limits in association with mortality. It is now established that the normal mPAP at rest is 14.0 ± 3.3 mmHg and the upper limit of normal mPAP in healthy individuals rarely exceeds 20 mmHg [47]. Additionally, several publications have shown that mPAP values from 20 to 25 mm Hg represent an independent predictor of poor survival [47,48].

Elevated PVR is associated with a higher mortality risk in PH patients [49]. The PVR threshold for precapillary PH was also recently adjusted from 3 to 2 wood units (WU) since this threshold was found to be the lowest prognostically relevant value [50]. In patients with PH associated with left heart disease or chronic obstructive pulmonary disease, mortality has been shown to increase continuously beginning at PVR 2 WU [51]. In PH associated with interstitial lung disease (ILD), PVR provides stronger prognostic information than mPAP alone [52]. In a retrospective cohort study among US veterans, Maron et al. identified the PVR threshold of 2.2 WU as clinically important in patients with high mPAP (≥19 mmHg) [49]. All-cause mortality and frequency of hospitalisation were significantly increased in this subgroup of patients. The authors found an adjusted hazard ratio for mortality of 1.71 (CI 1.59–1.84; *p* < 0.0001) among patients with an mPAP of ≥19 mmHg and PAWP of ≤15 mmHg. Effective PH management may improve PVR, preventing right ventricular (RV) failure and thus leading to a better prognosis. Moreover, Xanthouli et al. found out that in PAH-SSc patients, mPAP of 21–24 mmHg and PVR ≥ 2 WU were already associated with early pulmonary vascular disease, a decreased 6MWD and decreased tricuspid annular plane systolic excursion (TAPSE). In the same study, a PVR ≥ 2 WU was a significant predictor of reduced long-term survival (*p* = 0.002). Therefore, they proposed that a PVR threshold above 2 WU is more appropriate for mild PAH-SSc [53]. 

Elevated right atrial pressure (RAP) reflects RV pressure overload and is an established risk factor for mortality in PH. A poorer prognosis is demonstrated in PAH patients with high levels of RAP measured with the RHC. Benza et al. demonstrated that a mean RAP > 20 mmHg is an independent parameter of a worse prognosis [10].

Some studies have investigated the parallel performance of RHC and CPET. Weatherald et al. demonstrated that a decreased stroke volume index (SVI) and an increased RAP are independently associated with death or LT upon the first follow-up RHC in a large cohort of idiopathic, drug-induced and heritable PAH [45]. They also reported a superior predictive value of SVI over PVR, which directly reflects the pathologic increase in RV afterload. Comparing RHC and CPET in PAH patients, Pezzuto et al. demonstrated that peak end-tidal carbon dioxide tension (P_ET_CO_2_) > 26 mmHg and VE/VCO2 slope < 44 were associated with lower mPAP and PVR levels (*p* < 0.005). During follow-up of patients treated for 1 year for idiopathic, heritable or drug-induced PAH, the combination of a peak VO2 ≥ 15.7 mL/kg/min (≥60% predicted) and a cardiac index (CI) variation of ≥0.40 L/min/m^2^ may predict a low-risk patient status [54]. Furthermore, peak VO2 and SVI may provide further clinical information on intermediate-risk patients with idiopathic PAH and better predict outcomes [55]. In a multicentric study of the combination of RHC and CPET, low peak VO2, high PVR and increased heart rate during exercise (ΔHR) independently predicted poor prognosis in patients with idiopathic or familial PAH at 1 and 10 years [56].

Exercise PH was removed from the haemodynamic definition of PH after 2008 due to a lack of data defining a normal haemodynamic response during exercise. Several factors, independent from the pulmonary vasculature, may influence mPAP during effort such as patient age or hyperdynamic states. Several studies allowed a better definition of an abnormal mPAP/CO slope during exercise. Ho et al. demonstrated that the association of mPAP/CO slope > 3 mmHg/L/min and cardiovascular hospitalisation or all-cause mortality remained significant (*p* = 0.003), even after excluding resting PH [57]. Exercise PH was reintroduced in the 2022 guidelines and defined by an mPAP/CO slope > 3 mmHg/L/min between rest and exercise [9].

Mixed venous oxygen saturation (SvO2) is another prognostic parameter used by the 2015 ESC/ERS risk stratification guidelines [6] as it reflects oxygen extraction in the peripheral tissue and is correlated with CO. CO can be calculated by the equation: CO = VO2(oxygen uptake)/(SaO2 (arterial oxygen saturation)—SvO2)/(CO × Hb (haemoglobin level) × 1.34). This formula demonstrates that the SvO2 is directly associated with the CO and the oxygen delivery according to each patient’s need. However, it raises concerns about potential errors in measurement [29]. In a group of 98 patients with a CTEPH or PH associated with a systemic disease, Higgenbotam et al. demonstrated that SvO2 is a robust prognostic factor of survival [58]. However, the accuracy of SvO2 in predicting PH prognosis has not been consistently demonstrated in large cohorts.

### 2.4. Echocardiography and Cardiac Magnetic Resonance 

Echocardiographic parameters also have an important prognostic impact in PH patients [59]. Right atrium enlargement, reduced TAPSE and the presence of pericardial effusion have been shown to be associated with a worse prognosis. In a recently published study, TAPSE together with the degree of tricuspid regurgitation (TR) were significantly associated with TFS and proved to better stratify intermediate-risk patients [60]. For this group of patients, TAPSE < 19 mm predicted a worse prognosis with a 1-year survival of 74% compared to 96% in those with TAPSE ≥ 19 mm (*p* < 0.01). A moderate to severe TR also predicted a worse 1-year survival (70%) compared to no or mild TR (93%, *p* < 0.01). Combining those two parameters, intermediate-risk PAH patients with TAPSE < 19 mm and moderate/severe TR had an estimated 1-year survival (56%) similar to that of high-risk patients, whereas TAPSE ≥ 19 mm and no/trace/mild TR presented a 97% 1-year survival, similar to that of low-risk patients [60]. The latest finding underlines the central role of echocardiography among intermediate-risk PAH patients. Furthermore, in a retrospective multicentre French study, TAPSE/systolic pulmonary arterial pressure (sPAP) > 0.33 mm/mmHg appeared as a significant (*p* = 0.004) low-risk criteria for 3-year all-cause mortality or LT [61], reflecting right-atrio–ventricular coupling. Once added to the COMPERA score, the TAPSE/sPAP or TAPSE/regurgitation velocity ratio significantly dichotomised the intermediate-risk group into intermediate-low and intermediate-high subgroups [62]. In a recent study of the European Scleroderma Trials and Research cohort, a TAPSE/sPAP ratio < 0.55 mm/mmHg was a predictive risk factor for PH in SSc patients. A TAPSE/sPAP ratio ≤ 0.32 mm/mmHg was a predictive risk factor for all-cause mortality [63]. In another study, the TAPSE/sPAP ratio was an affordable and independent predictor for SSc-related cardiovascular events (*p* = 0.002) and mortality (*p* = 0.014). The combination of the TAPSE/sPAP ratio and amino-terminal atrial natriuretic peptide (NT-proANP) level may improve prognostic stratification in SSc (log rank *p* < 0.001) [64]. In addition, RV lateral free wall longitudinal strain (RVLS) and RV end-systolic dimensions represent newly validated and strong predictors of PH outcome [65].

Innovative noninvasive techniques were recently added to the ESC/ERS table for evaluation upon diagnosis, such as cardiac magnetic resonance (cMRI). CMRI is extremely useful for the assessment of RV morphology and function, which are critical in PH prognostic evaluation [66]. RV ejection fraction (RVEF), which reflects RV function, can independently predict 1-year mortality [67]. In a systematic review and meta-analysis, Alabed et al. showed the prognostic significance of CMRI, which can predict mortality and clinical deterioration in PAH patients with progressive RV dysfunction [68]. Predictive cMRI values upon a 1-year follow-up seem to be at least equal to that of RHC in a study conducted on 118 iPAH patients in the Netherlands [69].

## 3. Risk Stratification Scores and Decision of Lung Transplantation Referral

Several risk assessment tools have been validated from large PAH registry populations and have been shown to be extremely helpful in predicting survival by classifying patients into different risk score groups (low-risk, intermediate-risk, high-risk). The predictive value of REVEAL 2.0, ESC/ERS, COMPERA, FPHR and SPAHR scores has been extensively described in the literature [3,5,70]. Early identification of a patient’s risk status will provide a more accurate treatment approach with treatment escalation as needed in order to achieve a low-risk score and improve survival [71].

According to a survey conducted mainly in the United States and designed by the American College of Chest Physicians’ Pulmonary Vascular Diseases, less than 2/3 of the participants use those predictive scores during a patient’s evaluation [72]. Even fewer use them at follow-up appointments. However, more than half of the participants felt and understood that the score risk determination by these tools has changed the current management, which reflects their importance. These results could probably highlight the need for new scores with fewer parameters but with a strong prognostic value such as New York Heart Association (NYHA)/ WHO-FC, 6MWD and NT-proBNP [5]. The original COMPERA risk score and the abridged version of the REVEAL 2.0 risk score (REVEAL Lite 2) [73] are within the assessment tools with fewer parameters that are easily assessed in the outpatient clinic. However, in the case of missing variables, their predictive value is very limited [4]. 

The 2015 ESC/ERS guidelines separate patients into low, intermediate and high-risk groups to predict mortality at 1 year [6]. The majority of patients seen in PH centres were classified in the intermediate-risk group. Recently, redefined four-strata risk assessment scores were established from the French and the Swedish pulmonary registries, dividing the intermediate-risk group into intermediate-low and intermediate-high-risk [74,75] and incorporated in the 2022 ESC/ERS guidelines. Thus, by defining new variable cut-offs for each parameter (NYHA/WHO-FC, 6MWD, BNP or NT-proBNP), patients are now classified as low, intermediate-low, intermediate-high and high-risk. This new risk assessment method is better at predicting survival than the initial three-strata tools during follow-up. As a trade-off for convenient outpatient follow-up, this new risk assessment score does not include haemodynamic or echocardiographic characteristics, which may add accuracy to the prediction.

The REVEAL score was also modified over time to better classify mortality risk. In REVEAL 2.0, the number of parameters necessary to classify patients into different risk groups is modified compared to REVEAL 1.0. For example, a score ≤ 6 classifies PAH patients as low-risk, 7–8 as intermediate-risk and ≥9 as high-risk in the REVEAL 2.0 when compared to a score ≤ 8, 9 and ≥10, respectively, in the REVEAL 1.0 [73].

The 2022 ESC/ERS guidelines highlight the Importance of achieving a low or intermediate-low-risk group during follow-up [6]. It is now clearly recommended that achieving or maintaining an intermediate-high-risk profile does not represent an acceptable goal and should be considered inadequate. Consequently, for patients with an intermediate-high-risk profile during follow-up, a modification of treatment is required. Achieving or maintaining a low-risk profile during follow-up should represent the therapeutic goal for the vast majority of patients with PAH [5].

The role of echocardiography during follow-up is crucial and recommended in case of clinical deterioration or treatment modification. Fauvel et al. recently demonstrated that the three following criteria allow low-risk PAH patients to be identified upon follow-up: TAPSE/sPAP > 0.33 mm/mmHg, NYHA I-II and NT-proBNP < 300 ng/L or BNP < 50 ng/L [61]. Multiple echocardiographic measurements are significantly associated with survival in at least 90 days of parenteral prostacyclin [76]. Persistently severe or worsening RA dilation was strongly associated with outcome, highlighting the importance of the right heart evaluation in PAH [77].

It is essential to regularly re-evaluate patient risk status in order to identify a change in risk group and better guide management [70]. In a monocentric retrospective observational study conducted in Switzerland, a statistically significant improvement of the three risk assessment scores (COMPERA, REVEAL 2.0 and FPHR) after 1 year of treatment when compared to diagnosis was found for the transplant-free group [70]. This reflects the efficacy of the ongoing management and may guide clinicians either to downgrade therapy aggressiveness or to continue current medication. The significance of improvement endpoints was also highlighted by COMPERA: a sole improvement in 6MWD and NT-proBNP had minor prognostic value whereas improvements in multicomponent endpoints based on FC, 6MWD and NT-proBNP were associated with a better prognosis [78]. Recently, improvements in the four-strata risk assessment have been shown to predict outcomes in patients with iPAH, irrespective of the presence of comorbidities [79].

When maximal medical therapies fail to improve the risk score status, lung transplantation (LT) should be considered as it remains the ultimate therapeutic option in the most severe PAH cases. Therefore, it is crucial to define the right moment for LT referral and listing in order to reduce mortality and morbidity. Some PAH aetiologies, such as pulmonary veno-occlusive disease (PVOD), have a particularly poor prognosis and require prompt referral for lung transplantation. A persistent high-risk score implies worse outcomes, indicating that LT should be considered earlier in the case of PAH that remains refractory to triple combination therapy including a parenteral prostacyclin [80]. The use of the redefined four-strata risk assessment 6–12 months after iv treprostinil initiation has better classified intermediate-high-risk patients who were likely to benefit from LT [81]. High-risk patients have a 1-year mortality of >20%, which is notably higher than the 1-year mortality after LT, which is approximately 10% [82]. This means that clinicians should closely evaluate patients who are on triple combination therapy in order to carefully decide when they have the criteria for inscription on the LT waiting list. More evidence is however needed for intermediate-risk patients for potential LT listing [5,83]. Implementation of the lung allocation score (LAS) can help to prioritise patients on the LT waiting list, stratifying them into four groups according to their pulmonary disease [84]. Whereas LAS decreased waiting time, Russo et al. [85] published that recipients with high LAS have worse survival at 3 months and 1 year after LT than those with low LAS.

## 4. European and International PH Guidelines

As mentioned above, the 2022 ESC/ERS guidelines propose a three-strata model for risk stratification at diagnosis (low-, intermediate- and high-risk). Significant modifications are highlighted: NT-proBNP 1100 ng/L is now the new cut-off between intermediate- and high-risk patients (1400 ng/L in the 2015 guidelines). Additionally, TAPSE/sPAP is introduced for cardiac imaging: a ratio TAPSE/sPAP > 0.32 mm/mmHg classifies patients as low-risk, 0.19–0.32 mm/mmHg as intermediate-risk and <0.19 as high-risk. Parameters measured by the cMRI figure in the three-strata model include RVEF, SVI and right ventricular end-systolic volume index (RVESVI). Table 1 summarises the parameters and variables used for selected risk stratification models.

PAH treatment strategy and escalation are beyond the scope of this review. Briefly, recent guidelines recommend initiating, for patients of low or intermediate risk without comorbidities, an initial combination therapy with a combination of a phosphodiesterase type 5 inhibitor (PDE5i) and an endothelin receptor antagonist (ERA). For high-risk patients, initial combination therapy with a PDE5i, an ERA and a parenteral prostacyclin analogue should be considered [86].

A fundamental change in the recent guidelines consists of risk stratification during follow-up. As already mentioned, the four-strata model should be employed during follow-up, with evidence class I, level B, scoring patients as low-, intermediate-low-, intermediate-high- and high-risk. Comparing the two models, the four-strata better identifies intermediate-risk patients. In the COMPERA study, when the three-strata model was applied during the first follow-up, 31% changed their risk category. However, when the four-strata model was applied, 49.2% of patients changed their risk category, from whom 18.6% changed from the intermediate-low to intermediate-high-risk category [78]. The four-strata risk stratification model was also validated using the French PAH Registry. In an overall population of 2879 patients, using the four-strata model, a higher percentage of patients changed risk category upon follow-up (39%) compared to the three-strata model (29%). The four-strata risk model had a slightly higher prognostic value for long-term mortality and 1-year mortality compared to the three-strata risk model [74].

The role of RHC, at regular intervals during follow-up, in order to better define risk stratification could also be reassessed. The use of REVEAL 2.0 or the four-strata model, without the inclusion of invasive variables, could change practice and prolong the time to reconsider RHC in a more personalised approach [87].

In a state-of-the-art report from the American Thoracic Society, a complementary approach of several risk stratification models is recommended. The REVEAL 2.0 scale gives quantitative information that is useful during the discussion with the patient. The ESC/ERS scale gives a longitudinal therapeutic target taking into consideration multiple clinical and paraclinical examinations. The COMPERA registry points out the importance and simplicity of everyday information, that is WHO/FC, 6MWDT, NT-proBNP [88].

Concerning the latest international recommendations for CTEPH, echocardiographic variables that predict outcomes may be different compared to PAH. Parameters that reflect RV pressure overload, such as peak tricuspid regurgitation or RV acceleration time, seem more appropriate to evaluate the severity of CTEPH. On the other hand, those that indicate RV systolic function, such as TAPSE, are better to stratify PAH. In CTEPH, the appropriate selection of patients for different treatment modalities is of great importance and requires multidisciplinary team decisions. The careful choice between pulmonary endarterectomy (PEA), balloon pulmonary angioplasty (BPA), medical treatment or a combination of the modalities requires multidisciplinary discussion in expert centres. Long-term follow-up is necessary because of the risk of disease progression [89].

In paediatric PAH, a therapeutic strategy based on risk stratification and treatment response is recommended in children, extrapolated from that in adults, but adjusted for age [9,90]. The limit of mPAP in the definition of PH is also decreased in newborns after 3 months of age. The 2019 updated consensus statement proposed distinguishing paediatric patients as lower and higher risk. Predictors of a worse outcome are similar to those in adults. The choice of risk factor predictors also depends on the age of the child; for example, 6MWD is useful in children aged >6 years. NT-proBNP and uric acid have been shown to have prognostic value. Among the echocardiographic variables, TAPSE is the only strong predictor of survival. The intermediate-risk group is not specifically defined in children. This classification remains unchanged in the latest European consensus [90].

The new definition of PH has not yet been adopted by the Korean Society of Cardiology and Korean Academy of Tuberculosis and Respiratory Diseases and so they still use the Nice 2013 PH definitions [91]. This is not the case for China, where international guidelines are used [92,93]. Definitions were adapted according to the 6th WSPH in 2018.

The latest Japanese guidelines date back to 2017 and, consequently, the previous haemodynamic definition of PH is used. The 2015 ESC/ERS risk stratification model is incorporated in the Japanese guidelines [94]. Risk stratification strategies in Asian countries are therefore comparable to the ESC/ERS guidelines. For example, Teoh et al. recently reported a similar risk-stratification-based treatment strategy to the ESC/ERS guidelines in Singapore [95]. Table 2 summarises the selected risk stratification model(s) that the international guidelines have incorporated.

## 5. Conclusions and Perspectives

Risk stratification is an essential part of PAH patient management as outlined in the international guidelines and must be evaluated not only at baseline but also frequently during follow-up to adapt patients’ treatment and hence improve their prognosis. When patients remain at high risk despite maximal therapy, lung transplant referral or listing should be considered. Several parameters are already used as prognostic factors and new ones are currently evaluated for potential use in the future. PAH treatment should thus be tailored appropriately and in a personalised way in order to obtain and maintain a low-risk profile.

## Figures and Tables

**Table 1:** Parameters used in the different risk stratification models: the ESC/ERS PH Guidelines 2015 and 2022, the REVEAL 1.0 and 2.0 and the French PH Network Registry (FPHR). Grey background indicates the parameters included in the risk scores.

	ESC/ERS 2022	ESC/ERS 2015	REVEAL 1.0	REVEAL 2.0	FPHR
Hospitalisation					
eGFR					
Renal disease					
PAH aetiology					
PVR					
Male > 60 yrs					
SBP					
HR					
DLCO					
Pericardial effusion					
BNP					
NYHA class					
6MWD					
RAP					
Cardiac Index					
SvO2					
Clinical signs RHF					
Progressive symptoms					
Syncope					
RA area					
CPET					
TAPSE/sPAP					
cMRI					
Stroke volume index					

eGFR: estimated glomerular filtration rate; PAH: pulmonary arterial hypertension; PVR: pulmonary vascular resistance; SBP: systolic blood pressure; HR: heart rate; DLCO: diffusing capacity of the lungs for carbon monoxide; BNP: B-type natriuretic peptide; NYHA: New York Heart Association; 6MWD: 6-min walk distance; RAP: right atrial pressure; SvO2: mixed venous oxygen saturation: RHF: right heart failure; RA: right atrial; CPET: cardiopulmonary exercise testing; TAPSE/sPAP: tricuspid annular plane systolic excursion/systolic pulmonary arterial pressure; cMRI: cardiac magnetic resonance imaging.

**Table 2:** International and selected national recommendations with their respective PAH risk stratification models.

	Three-Strata Model	Four-Strata Model	Simplified Three-Strata Model	Lower and Higher Risk
ESC/ERS 2015	+	-	-	-
ESC/ERS 2022	Upon diagnosis	During follow-up	-	-
Japanese	+	-	-	-
Chinese	-	-	+	-
Pediatric	-	-	-	+

## Data Availability

Not applicable.
